# Transcutaneous Spinal Cord Stimulation: Advances in an Emerging Non-Invasive Strategy for Neuromodulation

**DOI:** 10.3390/jcm11133836

**Published:** 2022-07-01

**Authors:** Ursula S. Hofstoetter, Karen Minassian

**Affiliations:** Center for Medical Physics and Biomedical Engineering, Medical University of Vienna, 1090 Vienna, Austria

Recent studies of epidural electrical spinal cord stimulation have shown the enabling and, in some cases, the recovery of motor functions thought to be irreversibly lost due to severe spinal cord injury. These findings have marked the dawn of a new era in neurorehabilitation, in which unprecedented levels of improvements have become attainable even in the chronic stage of a lesion. With the development of transcutaneous spinal cord stimulation, a clinically accessible technique has complemented the current landscape of state-of-the-art neuromodulative therapeutic options. This method activates a subpopulation of the same neural structures of the spinal cord as engaged by epidural stimulation. As a non-invasive intervention, it holds the great potential to accelerate the wider application of electrical neuromodulation in the treatment of central nervous system diseases and injuries. Yet its firm establishment and lasting acceptance in clinical practice will not only hinge on the demonstration of safety and efficacy, but also on the delineation of the underlying physiological mechanisms. This will require an advance in our understanding of its immediate effects on neuronal circuits both in the intact and injured spinal cord. In parallel, there is a need to investigate clinical outcomes induced by repeated-administration regimens that go beyond the mere alleviation of symptoms. Importantly, such longer-lasting beneficial effects are indicative of structural and physiological plastic adaptations at various levels of the central nervous system. The present Special Issue is an ensemble of 16 contributions by 92 peers in the field. The articles shed light on the conceptual framework of the interplay between transcutaneous spinal cord stimulation and (altered) central nervous system function, seek to advance its usability, and explore untapped areas of application in neurorehabilitation following spinal cord injury.

A key question of studies focusing on improving lower-extremity motor function by spinal cord stimulation is to gain a better understanding about the integration of (residual) descending voluntary drive, externally applied electrical inputs, and the activity of sensorimotor circuits residing within the lumbosacral spinal cord. An important step in this direction is provided by Malloy and colleagues by introducing a clinically relevant rat model in which they adapted the procedure of transcutaneous spinal cord stimulation to activate neural targets and evoke short-latency spinal reflexes similar to those in humans [1]. In spinalized rats, their stimulation setup could be safely and stably applied over several weeks and with measureable modulatory effects on spinal reflex gain. The question of lumbar spinal sensorimotor integration is further addressed by Steele and colleagues [2]. In neurologically intact individuals, they used transcutaneous spinal cord stimulation as a non-invasive tool to assess spinal activation profiles during phases of preparation or execution of voluntary lower-limb motor tasks and demonstrated characteristic spatiotemporal patterns of increased or decreased spinal excitability. These findings are fundamental to the characterization of alterations in spinal-network function caused by injury or disease of the central nervous system. Such alterations are demonstrated by Calvert and co-workers in individuals with spinal cord injury [3]. During attempted voluntary movements of the lower limbs, spinal reflexes evoked either by epidural or transcutaneous spinal cord stimulation were significantly inhibited across muscles, irrespective of their functional role. Megía-García and colleagues studied the effects of single 10-min sessions of transcutaneous spinal cord stimulation applied at 30 Hz versus sham stimulation on lower-limb motor evoked potentials induced by transcranial magnetic stimulation in neurologically intact individuals [4]. They found increased amplitudes of motor evoked potentials in quadriceps during and after transcutaneous spinal cord stimulation, but not during or after sham stimulation. No effects on motor evoked potentials in tibialis anterior were observed. Their findings present an important interim step towards a better understanding of the spatial segmental effects of stimulation with associated clinical implications.

A comparatively new development in transcutaneous spinal cord stimulation necessitating basic mechanistic studies is its application over the cervical spinal cord with the aim to improve arm and hand function. Wecht and colleagues [5] investigated in individuals with and without chronic cervical spinal cord injury whether transcutaneous spinal cord stimulation paired with stimulation at other levels of the nervous system would enhance synaptic transmission in spinal circuits serving hand function. They found that subthreshold spinal stimulation magnified hand muscle responses to motor cortex stimulation, but not H reflexes and F waves induced by median nerve stimulation. Appropriately timed cortical and transcutaneous spinal cord stimulation may hence facilitate convergent sensorimotor transmission in the cervical spinal cord. The attainable neuromodulative outcomes of transcutaneous cervical spinal cord stimulation further depend on the applied stimulation intensity as well as on the level of voluntary participation in hand training, as shown by Kumru and co-workers in neurologically intact individuals [6]. Stimulation at 90% motor threshold led to higher maximum muscle grip strength, F-wave persistency, and maximum F wave to maximum M wave amplitude ratios, respectively, when compared to stimulation at 80 and 110% motor threshold. Stimulation at 90% motor threshold combined with training at 100% maximal volitional contraction in hand grip strength induced better hand function than with training at 50% maximal volitional contraction. The effects of sub-motor threshold transcutaneous cervical spinal cord stimulation were further examined by Sasaki and colleagues [7]. They studied whether stimulation applied for ten minutes in able-bodied individuals would alter corticospinal and spinal reflex activity at rest, yet found no modulation of motor evoked potentials or posterior root-muscle reflexes during or after the intervention. Likewise, McGeady and colleagues, who applied cervical stimulation for 10 min at the individually maximum tolerable intensity, found no consistent alterations in cortical oscillatory dynamics across their cohort of neurologically intact participants [8]. However, a weak inhibitory effect at cortical level was observed in those individuals who received the stimulation at the highest intensity levels.

While transcutaneous spinal cord stimulation is a clinically accessible method, a few aspects need to be considered to avoid potential pitfalls that could negatively impact its efficacy as a therapeutic intervention. This is partially because the stimulation conditions are sensitive to biophysical changes in the volume conductor in-between the surface electrodes that determine the electric field acting on the targeted neural structures. Binder and colleagues addressed this issue by studying the effect of extended, neutral, and flexed spine alignments in different body positions on the elicitation of H reflexes and posterior root-muscle reflexes in neurologically intact individuals [9]. They showed that, with neutral or extended spine alignments, the target neural structures of transcutaneous spinal cord stimulation in the posterior roots were most reliably activated and recommend body positions that allow easy maintenance of such alignment for therapeutic applications. Technological developments to further ease the use of transcutaneous spinal cord stimulation in clinical environments are a necessary prerequisite for its wide acceptance and lasting integration into rehabilitation practice. Salchow-Hömmen and colleagues tackled this question by introducing a novel algorithm that allows to automatically calibrate the stimulation setup and determine required stimulation intensities for antispasticity applications for each individual treated, all within a few minutes only [10]. The spasticity-alleviating effect of transcutaneous spinal cord stimulation as a viable non-pharmacological approach was further investigated by Sandler and colleagues [11]. In a randomized crossover trial including 32 individuals with motor-incomplete spinal cord injury, they tested the effects of single sessions of either transcutaneous spinal cord stimulation or whole-body vibration. Both methods reduced quadriceps spasticity for time periods beyond the intervention. Estes and colleagues combined transcutaneous spinal cord stimulation and locomotor training in individuals with sub-acute motor-incomplete spinal cord injury to enhance walking function and alleviate spasticity and compared the results to a paradigm employing sham stimulation and locomotor training [12]. In the verum group, walking outcomes were significantly improved after the intervention period of two weeks. No alterations in spasticity were seen in either group, which was partially attributed to the variability in spasticity encountered in the study participants. The influence of transcutaneous spinal cord stimulation on voluntary movement and locomotion in chronic motor-incomplete spinal cord injury were further addressed by Meyer and colleagues by studying the immediate effects of stimulation applied at different frequencies [13]. They found an increased maximum dorsiflexion angle and range of movement during rhythmic ankle movements of the more affected lower limb with 30 Hz stimulation compared to baseline, but not with 15 or 50 Hz stimulation. Electrophysiological assessments further showed a significant reduction of pathological components of polysynaptic spinal reflexes during stimulation at 30 Hz. The effects on walking were variable, with improvements seen in the subject with the highest walking scores as well as in a subgroup of the participants with the lowest locomotor function. Al’joboori and colleagues studied the outcomes of an 8-week sit-to-stand training paradigm with and without transcutaneous spinal cord stimulation in a small cohort of individuals with motor-complete or incomplete spinal cord injury [14]. While unassisted standing was not achieved in any participant, motor scores were improved, and volitional lower-limb movements were partially recovered in three individuals in whom the training had been complemented by stimulation. No such changes were observed in the control group. The importance of rehabilitation paradigms combining training and electrical stimulation is also highlighted by the work of Kumru and co-workers, who targeted the cervical spinal cord to enhance hand function [15]. Single sessions of transcutaneous cervical spinal cord stimulation applied during hand training in neurologically intact individuals retained hand grip force and increased spinal and corticospinal excitability for at least an hour. Stimulation alone increased spinal but not corticospinal excitability and had no effect on hand grip force, and training alone reduced both hand grip force and corticospinal excitability.

The Special Issue is rounded off by a review contributed by Barss and colleagues [16], in which they trace the development of transcutaneous spinal cord stimulation as a neuromodulation intervention after spinal cord injury. They elaborate on the efficacy of the stimulation applied to distinct levels of the spinal cord to induce multi-segmental effects and on how multi-site stimulation may facilitate spinal reflex and corticospinal network activity. The review provides an overview of current potentials and limitations of transcutaneous spinal cord stimulation directed to both the cervical and the lumbar spinal cord.
